# Global Trends of Green Pesticide Research from 1994 to 2019: A Bibliometric Analysis

**DOI:** 10.1155/2021/6637516

**Published:** 2021-03-19

**Authors:** Hein Hendrik Smith, Oladayo Amed Idris, Mark Steve Maboeta

**Affiliations:** Unit for Environmental Sciences and Management (UESM), Faculty of Natural and Agricultural Sciences, North-West University, Potchefstroom, North-West, South Africa

## Abstract

The fast-growing world population places food production under enormous pressure to ensure food security. One of the most common methods to increase food production is the use of pesticides, but the continuous use thereof has numerous detrimental effects on the environment. The interest in biopesticides for a possible substitute has grown over the past two decades. To determine the research evolution of biopesticides (green pesticides), a bibliometric analysis from 1994 to 2019 was carried out. A total of 580 documents were found eligible in the Scopus database for this analysis. Parameters such as the number of articles, article citations, keywords, source impact, and countries of publication were used to analyse the documents and rank countries based on authors, productivity, article citations, and co-authorship. The analysis reveals production increased significantly from 2009 and has the most published documents in 2019 with a total of 74 articles. Asia's most populous countries, India and China, were ranked first and second, respectively, and the USA third in terms of the most productive countries in the field of plant biopesticides. Countries in Europe and Africa however have fewer publications than expected in this field, given the fact that they are high consumers of pesticides. India, China, and the USA have 4.08%, 2.94%, and 12.5% multiple country publications (MCPs), respectively, with the USA having a stronger collaboration. Finally, there is a clear indication in this study that India and China are taking the lead in substituting synthetic pesticides with the alternative natural plant biopesticide.

## 1. Introduction

The detrimental effects of synthetic pesticides on the environment are well established as it affects animal and plant biodiversity as well as terrestrial and aquatic ecosystems [1]. Other effects include toxicity, soil and groundwater pollution, and harmful residues that contaminate crops and food. It also affects non-target organisms and the excessive use of synthetic pesticides leads to increased resistance in pests [2]. The negative effects of synthetic pesticides have created the need for a safer and more environmentally friendly substitute. Biopesticides are believed to be less toxic, environmentally friendly, and not harmful to humans and non-target organisms [2]. The use of botanical insecticides is not a new practice. As early as the 17th century, botanical insecticides have been used in agricultural practices by ancient China, Egypt, Greece, and India [3]. Over the past decade, the interest in biopesticides has grown immensely, with the intention to substitute with synthetic pesticides. According to a study done by Isman and Grieneisen [4], the number of annual papers published on botanical insecticides has grown from 61 papers in 1980 to 1207 papers in 2012 [4]. This signifies an increase in the awareness and uses of plant biopesticides. In spite of the growing need for natural pest management, plant biopesticides contribute less than 1% of the total use of pesticides [2]. The annual amount of synthetic pesticides used for crop protection is estimated at 2.5 million tons causing damages worth about $100 billion, due to residues in crops, soil and water, nonbiodegradable properties, and high toxicity [5]. Synthetic pesticides are no doubt a threat to public health and demand an urgent alternative with better crop protection and less harmful properties. The term “green pesticides” entails all naturally acquired types of pest management from plant extracts, plant-derived pesticides, plant secondary metabolites, and plant-based pesticides [5]. The green pesticides are also referred to as botanical pesticides and are used as repellents, nematicides, insecticides, fungicides, and bactericides, obtained in the form of isolated substances or complex mixtures [6]. Although there has been significant growth in botanical pesticide research, the commercial availability of these pesticides remains limited [7]. According to Isman and Grieneisen [4], there are a few limitations of the exploitation of botanical pesticides such as lack of availability of good quality plant pesticides at affordable prices, strict regulation particularly on endangered plant species, short persistence of the phytochemical in the environment, and the extraction of the phytochemical from plants grown under different climatic conditions resulting in different compositions in terms of active agents, which leads to variability in effectiveness [4]. One of the main factors preventing the advancement of commercializing botanical pesticides is sustainability [6] of the product. In order to produce botanical pesticides on a commercial scale, it is necessary to obtain large amounts of the source plant, which requires large scale production [6]. However, most biopesticide plants are still not grown commercially, making it difficult to source for the plants. Although research on botanical pesticides has increased significantly over the past two decades [8], the above-mentioned limitations still contribute to the lack of commercialization of botanical pesticides.

With the continuously growing interest in botanical pesticides, it is necessary to carry out a bibliometric compilation to better understand the research trend. The fast-growing number of academic publications makes it extremely difficult to stay up to date with everything being published [9]. To help organise and better understand previous research, different quantitative and qualitative literature reviewing approaches are used. Bibliometrics is one such approach and according to Aria and Cuccurullo [9] it has the potential to perform a “systematic, transparent, and reproducible review process based on the statistical measurement of science, scientists, or scientific activity.” Various research fields make use of bibliometrics to measure the impact or influence of certain research articles. For example, in the field of engineering, the authors Bao et al. [10] used bibliometric analysis to evaluate the publications in the field of robotic exoskeletons and the authors He et al. [11] used bibliometrics to analyse articles related to “metastatic castration-resistant prostate cancer” in the field of health and medicine. Muhuri et al. [12] projected the growth structure of the ongoing industrial revolution “Industry 4.0” using bibliometric analysis in the field of intelligent engineering [12]. All these examples mentioned but few indicate bibliometric study is relevant in many fields.

During this study, the objective was to analyse the global research activities on botanical pesticides from 1994 to 2019, in the Scopus database. The data obtained was then used to study the research progression using bibliometric parameters and indicators such as most productive authors, most productive countries, annual scientific production, country collaboration, average article citations per year, average total citations per year, most relevant keywords, bibliographic coupling, and documents production source impact. The Scopus database was chosen as it includes a wider spectrum of journals in citation analysis and keyword searching than PubMed and Web of Science [13]. It also offers an easy trajectory through the extrapolation of all the bibliometric indicators proposed in this study. The period 1994–2019 was chosen based on the fact that previous studies indicated that research on green pesticides has only become of increasing interest over the past two decades, due to increase in pesticides and agrochemicals residues which are causing significant contamination in the environment [14].

Due to the detrimental effects of synthetic pesticides on both the environment and human population, this study aims to carry out a descriptive bibliometric analysis on green pesticide research from 1994 to 2019, in order to present a general overview of global research trends towards finding an alternative for synthetic pesticides.

## 2. Materials and Methods

The published research outputs on botanical pesticides were retrieved from the Scopus database on 7 August 2020. The Scopus database provides access to 27 million abstracts with citations dating back to 1966 [15]. The key terms used to query Scopus database in this study were “plant pesticides”, “plant biopesticides”, “plant-derived pesticides”, “green pesticides”, “botanical pesticides” and “phyto-pesticides”, using the Boolean operator “OR”. These key terms were used to retrieve title-specific research from 1994 to 2019 which returned 586 documents. The documents were cleaned and downloaded in BibTex file format [16] and then analysed for a bibliometric statistics with R-project version 4.0.2. The codes used to extract the bibliometric indices were in accordance with Aria and Cuccurullo [9] in Rstudio interface. The bibliometric indices, annual scientific production, article citation per year, bibliographic coupling, most productive authors, most productive countries, most relevant keywords, and most productive journals/source, were used in this study to evaluate the current and possible future trends of the research in plant biopesticides. In this study, the annual scientific indicator is tallied up by institute, organisation, author, and countries. The citation analysis measures the relative importance or impact of an author or publication has been cited by others. However, the citation counts were used to measure the impact per country on plant biopesticides. The publication-citation matrix was used to analyse most productive authors/countries, co-citation, and bibliographic coupling as described [9, 17]. The keywords extracted directly from the documents with the R-project interface are used to study the conceptual structure of the green pesticide research field.

## 3. Results

### 3.1. Green Pesticide Research Production

**Figure 1:**
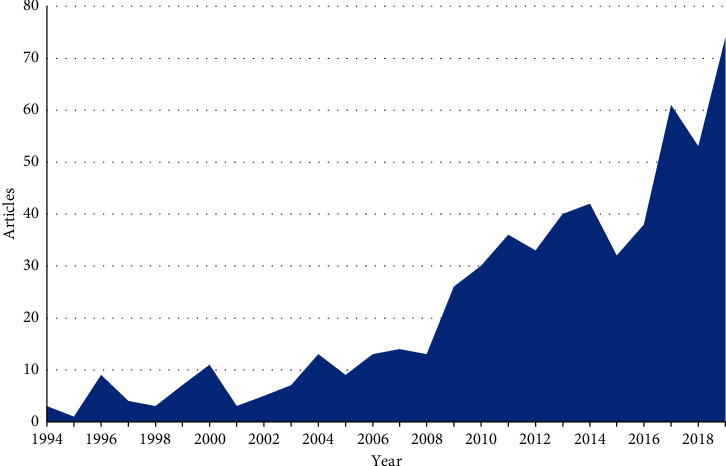
Annual scientific publications of documents on plant biopesticides from 1994 to 2019 in the Scopus database.

To determine the progression of research on green pesticides in this study, a wide spectrum of biopesticide keywords were used to query the database. A total of 540 published documents, from 332 sources, were recorded on green pesticides. These documents include articles, books, book chapters, conference papers, conference reviews, editorials, erratum, notes, reviews, and short surveys. An average citation of 12.63 per document makes them relevant in the scientific community (Table 1). The annual trend of scientific production from 1994 to 2008 showed a minimal increase, with the highest increases in 2000, 2004, and 2007 (Figure 1). Production increased significantly from 2009, with the most documents published in 2019 (74 documents). A decline in publications, however, occurred during 2015 and 2016, with 32 and 38 publications, respectively. The reason for such a decline is not known. The annual percentage growth rate from 1994 to 2019 was 13.68%. It is also worth noting that although there is a steady increase in the annual scientific production, fluctuations do occur between some years. Possible explanations could include improved laboratories, the evolvement of new researchers, the development of new research methodology, and funding support [18].

### 3.2. Keywords on Green Pesticide Research

**Figure 2:**
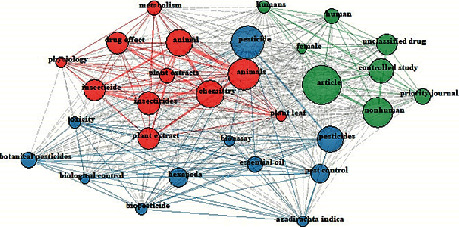
Keyword co-occurrences network of the top 30 keywords on green pesticide research. The circle size of the keywords represents the frequency of occurrence in articles. The thickness of the line between any two keywords displays the degree of cooperation.

Most published documents contain a number of keywords to assist with online searches and identify certain editors to a document [18]. To study the research trends of green pesticides, both the singular and plural form of the author keywords were used. This aids in understanding the research evolution of the study field [19]. Both the author keywords and keyword plus were included in the study. The author keywords are a list of terms provided by the author/s that best represents the content of the document, while keyword-plus are terms or phrases that occur in the titles of a document's references but do not appear in the document's title itself [20]. Between 1994 and 2019, a total of 1732 author keywords and 4064 keyword-plus terms were retrieved from the documents on green pesticide research (Table 2). The most used author keyword was botanical pesticide/s occurring in 159 articles, followed by essential oil/s (41 articles) and pesticide/s (25 articles), second and third, respectively. Other author keywords occurring in the top 30 include green pesticides (22 articles), biopesticides (17 articles), pest management (14 articles), toxicity (12 articles), and plant extracts (13 articles) etc.; all these are relevant to pest control using plant biopesticides. The most used keyword-plus was animal/s, occurring in 220 articles, followed by pesticide/s (217 articles) and insecticide/s (175 articles), second and third, respectively, as shown in Table 2. It is not surprising to get a broad range of terms in keyword-plus because it is derived from the titles of documents cited by the author. Other keyword-plus terms in the top 30 are plant extract (102 articles), pest control (82 articles), essential oil (62 articles), and toxicity (38 articles). Although it is presumed that green pesticides are harmless to the environment, it is important to note that the keyword toxicity is listed in the top 30 of both the author keywords and keyword-plus terms, which could be referring to the potency of biopesticide plants. Most biopesticide plants are toxic but have a very short half-life [21]. This could have contributed to the general belief that plant biopesticides are natural and therefore harmless, which is not always the case. In Figure 2, the co-occurrence network and interrelationship of the top 30 terms on green pesticide research are represented with a pictographic network. Each coloured circle represents a cluster of terms and the connecting lines represent the cooperation degree. Pesticide related terms are in blue clusters and the response related terms in red clusters (Figure 2).

### 3.3. Productivity on Green Pesticide Research per Country

**Figure 3:**
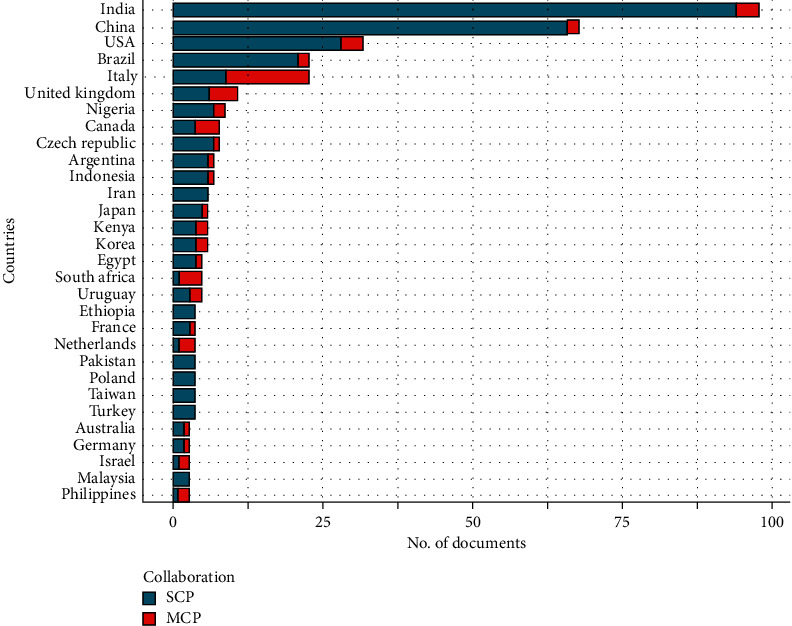
Most productive countries and collaboration in the research of green pesticides from 1994 to 2019. SCP: Single Country Publications, MCP: Multiple Country Publications.

India is ranked first in the most productive countries with a total of 98 publications, among which 4.08% are published out of collaboration. China and USA are ranked second and third with 68 and 32 publications, respectively. China has 2.94% and USA has 12.5% multiple country publications (MCP) which indicate the USA collaborates more while China relies more on local research. It is quite interesting to know that Italy and South Africa have fewer single country publications (SCP) compared to their MCP; hence they have a higher MCP: SCP ratio. The collaboration on publications of Italy, Netherlands, and South Africa is 60.86%, 75.0%, and 80.0%, respectively, making these the countries with the most collaboration network (Figure 3).

Table 3 shows that India is ranked first (1080 citations; h-index 18), followed by USA (979 citations; h-index 17) and Italy (868 citations; h-index 16), second and third, respectively. African countries in the top 30 list are Egypt in 8th (184 citations), South Africa 12th (92 citations), Zimbabwe 13th (90 citations), Nigeria 15th (69 citations), Kenya 20th (54 citations), and Tanzania 22nd (52 citations). It is debated that citations reflect the scientific impact and relevance of an article, but with limitation to the measures of the research quality [22, 23]. Hence, the citation ranking the USA first and other countries following could be the measure of their study relevancy. It is important to note that Europe is the largest consumer of pesticides with Asia the second largest and the leading countries in pesticide production are China, USA, France, Brazil, and Japan [24]. In Figure 3 and Table 3, the above-mentioned countries are ranked in the top 30 of the categories of the most productive and most cited country in plant biopesticide study, but with the exception of Japan, indicating the countries are focusing on plant biopesticides as an alternative to synthetic pesticides, and these studies are relevant in the scientific community [25].

**Figure 4:**
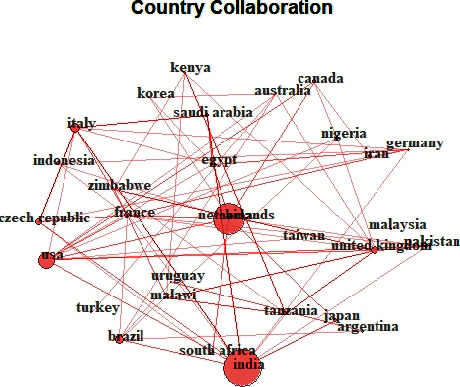
Country collaboration network of the top 30 most productive countries on green pesticide research. Line thickness represents the collaboration strength between countries.

According to Lee and Bozeman [26], scientific collaboration in research has become the standard. Modern science is becoming ever more interdisciplinary and complex, which encourages research collaboration. Many funding agencies encourage collaborative research by incorporating this as one of their funding conditions [26]. The top 30 country collaboration network on green pesticides is shown in Figure 4. The coloured circles represent the countries and the circle size represents the number of collaborations with other countries. Thicker lines between countries illustrate collaborative strength between countries. As revealed by the bibliometric analysis, Italy, United Kingdom, USA, Canada, South Africa, India, and Netherlands have the most collaborations with other countries. Figure 4 shows the frequency and network of collaboration between the countries. India, United Kingdom, Netherlands, Brazil, Canada, and the USA collaborate often. Italy, Czech Republic, Saudi Arabia, and India are regular collaborators. And Netherlands, South Africa, and the USA also frequently collaborate, all these forming a network. The collaborative strength of some of these countries could be ascribed to the respective countries history of pesticide use and government funding encouraging collaboration [18].

### 3.4. Most Cited Documents on Green Pesticides and Source Impact

Table 4 presents the most cited papers on plant biopesticides. The top-ranked article published by Pavela and Benelli [27] had a total of 288 citations at an average of 57.6 citations per year. In the article, the strengths, challenges, and constraints of essential oil-based biopesticides were analysed [27], considering the effectiveness, toxicity, and the mechanisms of action. The high citation accredited by the article may be due to the wide spectrum of the biological activity such as nematicidal, ovicidal, fungicidal, insecticidal, and bactericidal effect of essential oil-based biopesticides making the article relevant in a wide field of research. Surprisingly, only 3 articles from the 1990s are listed in the top 30 most cited documents. This may indicate that more recent researchers are becoming more interested in plant biopesticides, as search for alternative synthetic pesticides grows.

**Figure 5:**
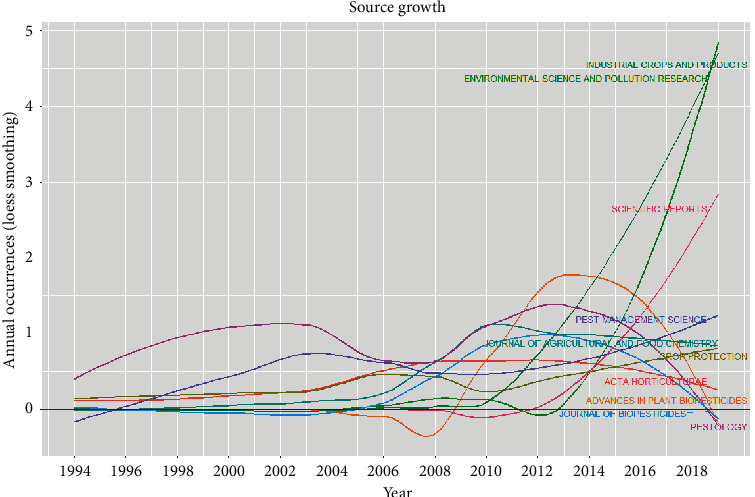
Source growth of the top 10 most productive journals from 1994 to 2019.

In Table 5, the top 30 productive journals in terms of the number of publications, total citations, and h-index are reported. The top 30 journals have published 219 articles which represent 37.76% of the documents in this study. The Pestology journal is ranked first (NP: 24; h-index; 4), Industrial Crops and Products second (NP: 21; h-index: 12), and Pest Management Science third (NP: 14; h-index: 10). The source growth of the top 10 productive journals is shown in Figure 5. The journals “Environmental Science and Pollution Research,” “Industrial Crops and Products,” and ‘Scientific Reports” have grown exponentially over the last few years. On the contrary, the impact of the journals “Pestology,” “Acta Horticulture,” and “Advances in Plant Biopesticides” has drastically reduced in the field of plant biopesticides over time. It is also noteworthy that 25 years ago very few journals published articles on green pesticides. Recently, there are more publications on green pesticides indicating that the research area is growing.

## 4. Discussion

The increase in publications from 2009 could be due to the increasing realization of the detrimental effects of synthetic pesticides on the environment [1], the growing need for a sustainable alternative, government policies on environmental protection, and pressure groups [56].

To determine the productivity of a research field based on a bibliometric study, the number of scientific publications is investigated [57]. Globally, demand for biopesticides has increased due to rising interest in organic products as a potential alternative to synthetic insecticides [58]. The demand could have driven the raising research and publication in the field of plant biopesticides as shown in Figure 1. Andreo-Martínez et al. [59] in their study reported an increase of publication related to pesticides bioavailability in food, vegetables, and wine from 1 in 1976 to 154 in 2018 [59]. This is in accordance with our study as there is a steady and sharp increase in the publication of biopesticides related work from 2009. A change in the number of publications in a research field could indicate a possible change in demand and technology. In Table 2, the keyword-plus “animal/s” is ranked first. This could be due to the increasing demand and use of plant phytochemicals for ectoparasites on livestock [60]. The use of plant biopesticides is common especially among rural areas communities that are dependent on livestock for survival. Aside from the cultural practices, these communities often do not have the financial capability to buy pesticides and instead make use of biopesticides for pest management [60].

Considering the data collected in this study as shown in Table 1, a total of 580 documents from 332 sources were retrieved from the Scopus database. These documents were authored by 1867 researchers, on green pesticides from 1994 to 2019 with an estimate of 0.311 documents per author and 3.22 authors per document. Out of the 1867 authors, 1799 were authors of multi-authored documents with the remaining 68 being authors of single-authored documents. The average number of authors per document is referred to as collaboration index [61]. Research collaboration and country collaboration network as shown in Figures 3 and 4 of this study are the bibliometric indicators that reflect the social structure among countries, institutions, and researchers. Many previous bibliometric studies in different fields ranked the United States as being dominant in international collaboration [62, 63]; on the contrary, our analysis reveals Italy dominates international collaboration on green biopesticide followed by the United Kingdom. International research collaboration allows a synergy of knowledge, idea, and research fund that enhances the quality of research. Collaboration has a great impact on the citation of a document; particularly internationally co-authored papers were reported to receive twice the average citation as local co-authored papers [64]. India, however, tops the most cited countries on green biopesticide papers and most of these countries in Table 3 are also reported in the previous bibliometric study of pesticides [59]. The collaboration index (CI) for green biopesticide as established in this study is 3.59, which is considered as high participation of co-authorship [61]. A study by Liu et al. [24] on global biodiversity research reported the worldwide research collaboration index to be 4.45 [24], which is higher than the CI reported in this study. The CI of global biodiversity research as reported could be the ripple effect of climate change which is a major driver of biodiversity [65].

In Figure 3, India and China are ranked first and second, respectively, in green pesticide research; this may be attributed to their long history of the use of botanical insecticides [3]. The African continent has 5 countries in the top 30 most productive countries: Nigeria (7th), Kenya (14th), Egypt (16th), South Africa (17th), and Ethiopia (19th) with 9, 6, 5, 5, and 4 publications, respectively. Although Africa consumes less than 5% of global pesticides, most countries in Africa still use the most toxic pesticides in food production [66]. According to Dinham [67], small-scale farmers apply these toxic pesticides weekly, sometimes more often, during the growing season [67]. This raises a number of concerns to both environmental and human health experts in Africa. Finally, the above-mentioned factors indicate that African countries are not doing enough in pesticide regulations and the research towards an alternative which means the continent still needs to do more in pest management while jacking up crop production.

The limitations that arose during this study were language, the fact that the quality of published documents could not be ascertained, and the research not available on Scopus being missed out. The documents retrieved from the Scopus database were limited to “English written documents.” Documents published in other languages also contribute to research productivity but were unfortunately eliminated in this study. The quality of published documents cannot be measured by total citations alone while the annual scientific production of an author or a country is also not a measure of research quality [18]. It is worth noting that bibliometric analysis does not criticize any part of a research document; hence quality control could be problematic. Regardless of the limitations associated with this study, it provided a global overview of green pesticide research productivity from 1994 to 2019.

## 5. Conclusion

This bibliometric study discussed the global trend of green pesticide research from 1994 to 2019 based on documents retrieved from Scopus. Despite the increasing research on green pesticides, commercial applications remain limited. Trends in the annual scientific production suggest that research on green pesticides will continue to increase. The detrimental effects of synthetic pesticides on the environment and human health could be the possible drivers for research productivity in the alternative field of plant biopesticides. This study also found that India, China, and USA are the leading countries in the production of green biopesticide research documents articles and amass the highest total citations per country. Countries in Europe and Africa, although high consumers of pesticides, have significantly less published research documents compared to the earlier mentioned countries. Europe and Africa countries need to intensify their research on alternative pesticides through either government or privately funded programs to promote environmentally friendly pesticides, especially in Africa where pesticides with high toxicity are still being used.

## Figures and Tables

**Table 1:** Summary of data retrieved from Scopus database.

Description	Count
Documents	540
Source (journals, books, etc.)	332
Keywords plus (ID)	4064
Author's keywords (DE)	1732
Period	1994–2019
Average citations per document	12.63
Authors	1867
Author appearances	2408
Authors of single-authored documents	68
Authors of multiauthored documents	1799
Single-authored documents	79
Documents per author	0.311
Authors per document	3.22
Co-authors per document	4.15
Collaboration index	3.59

**Table 2:** Top 30 most relevant keywords on green pesticide research from 1994 to 2019.

Rank	Author keywords (DE)	Articles	Keyword plus (ID)	Articles
1	Botanical pesticide/s	159	Animal/s	220
2	Essential oil/s	41	Pesticide/s	217
3	Pesticide/s	25	Insecticide/s	175
4	Azadirachtin	24	Plant extract/s	151
5	Green pesticides	22	Article	117
6	Biopesticides	17	Chemistry	90
7	Neem	15	Pest control	82
8	Pest management	14	Hexapoda	79
9	Botanical insecticide/s	14	Nonhuman	78
10	*Azadirachta indica*	13	Drug effect	69
11	Botanicals	13	Azadirachta indica	65
12	Plant extracts	13	Essential oil	62
13	*Spodoptera litura*	12	Botanical pesticides	60
14	Toxicity	12	Controlled study	57
15	Mortality	11	Larva	50
16	Integrated pest management	10	Biopesticide	49
17	Antifeedant	9	Unclassified drug	43
18	Biological control	9	Female	42
19	Insecticidal activity	9	Lepidoptera	39
20	Repellency	9	Metabolism	38
21	Bioassay	8	Toxicity	38
22	Contact toxicity	8	Human	37
23	Insecticide	8	Bioassay	35
24	Pest control	8	Agriculture	33
25	Aphids	6	Biological control	33
26	*Heteropneustes fossilis*	6	Insect	32
27	Phytochemicals	6	Mortality	32
28	*Plutella xylostella*	6	Fungi	31
29	*Tetranychus urticae*	6	Moth	31
30	Acute toxicity	5	Physiology	27

**Table 3:** Citations per country in terms of green pesticide research from 1994 to 2019.

Rank	Country/region	Total citations	Average article citations (%)	MCP ratio	h-index
1	India	1080	11.020	0.041	18
2	USA	979	30.594	0.125	17
3	Italy	868	37.739	0.609	16
4	China	682	10.029	0.029	14
5	United Kingdom	441	40.091	0.455	11
6	Brazil	389	16.913	0.087	10
7	Canada	251	31.375	0.500	9
8	Egypt	184	36.800	0.200	7
9	Argentina	162	23.143	0.143	7
10	Belgium	105	52.500	1.000	6
11	Czech Republic	94	11.750	0.125	5
12	South Africa	92	18.400	0.800	5
13	Zimbabwe	90	45.000	1.000	5
14	Iran	86	14.333	0	5
15	Nigeria	69	7.667	0.222	4
16	Korea	68	11.333	0.333	4
17	Uruguay	64	12.800	0.400	4
18	Turkey	59	14.750	0	4
19	Israel	55	18.333	0.667	4
20	Kenya	54	9.000	0.333	4
21	Netherlands	52	13.000	0.750	4
22	Tanzania	52	17.333	0.667	4
23	Poland	44	11.000	0	3
24	Romania	44	44.000	0	3
25	Australia	42	14.000	0.333	3
26	Taiwan	39	9.750	0	3
27	France	38	9.500	0.250	3
28	Greece	38	38.000	0	3
29	Germany	27	9.000	0.333	2
30	Lebanon	27	27.000	0	2

MCPs: multiple country publications.

**Table 4:** The top 30 most cited documents on green pesticide research from 1994 to 2019 from Scopus.

Rank	First authors	Journal	TC	TC/year
1	Pavela Roman, 2016	Trends Plant Sci	288	57.6
2	Mulla S. Mir, 1999	J Am Mosq Control Assoc	242	11.0
3	Morgan E. David, 2009	Bioorg Med Chem	161	13.42
4	Son T. Gen, 2010	J Neurochem	135	12.27
5	Miresmailli Saber, 2014	Trends Plant Sci	134	19.14
6	Dodge Jeffrey A, 1996	J Steroid Biochem Mol Biol	130	5.2
7	Benelli Giovanni, 2017	J Cluster Sci	101	25.25
8	Attia Sabrine, 2013	J Pest Sci	99	12.37
9	El-Wakeil Nabil E, 2013	Gesunde Pflanz	95	11.87
10	Qian Xuhong, 2010	J Agric Food Chem	89	8.09
11	Cresswell James E, 2012	Pest Manage Sci	71	7.89
12	Sibanda T, 2000	Crop Prot	71	3.38
13	Mossa Abdel Tawab H, 2016	J Environ Sci Technol	68	13.6
14	Koul Opender, 2009	Cab Rev Perspect Agric Vet Sci Nutr Nat Resour	68	5.67
15	Kotkar Hemlata M, 2002	Pest Manage Sci	68	3.58
16	Charleston Deidre S, 2005	Biol Control	64	4.0
17	Martin Krista M, 2000	J Am Vet Med Assoc	64	3.05
18	Benelli Giovanni, 2017	Parasitol Int	62	15.5
19	Hunt Piper R, 2011	Plos One	62	6.2
20	Dubey NK, 2010	Curr Sci	61	5.55
21	Zheng Ke, 2012	Acta Chim Sin	60	6.67
22	Mann Rs, 2012	Mini-Rev Org Chem	59	6.56
23	Rao K Jagajjanani, 2013	Rsc Adv	56	7.0
24	Vidal Estrela Joelma Lima, 2006	Pesqui Agropecu Bras	54	3.6
25	Benelli Giovanni, 2018	Ind Crops Prod-A	53	17.67
26	Caboni Pierluigi, 2012	J Agric Food Chem	51	5.67
27	Zibaee A, 2010	Bull Entomol Res	51	4.64
28	Moreira Márcio D, 2007	Pest Manage Sci	51	3.64
29	Mansour F, 2004	Phytoparasitica	50	2.94
30	Valladares G, 1997	J Econ Entomol	49	2.04

TC = total citations.

**Table 5:** Top 30 productive journals, the total number of publications, total citations, h-index, and publication start year, from 1994 to 2019.

Source	NP	TC	h-index	PY-Start
Pestology	24	48	4	1996
Industrial Crops and Products	21	334	12	2010
Pest Management Science	14	378	10	2000
Environmental Science and Pollution Research	13	155	6	2010
Advances in Plant Biopesticides	13	91	7	2014
Journal of Agricultural and Food Chemistry	12	342	8	2003
Crop Protection	10	258	9	1997
Acta Horticulturae	10	20	2	1997
Scientific Reports	9	75	5	2016
Journal of Biopesticides	8	28	4	2010
Plos One	7	150	5	2006
Journal of Pest Science	6	172	6	2009
Molecules	6	39	4	2010
Green Pesticides Handbook: Essential Oils for Pest Control	6	5	2	2017
ACS Symposium Series	5	50	2	1996
Journal of Pesticide Science	5	23	3	2009
Biopesticides International	5	5	2	2011
Phytoparasitica	4	85	3	2004
Pesticide Biochemistry and Physiology	4	54	3	2011
Journal of Insect Science	4	28	2	2014
Archives of Phytopathology and Plant Protection	4	16	2	2011
Zhongguo Zhongyao Zazhi	4	7	2	2004
IOP Conference Series: Earth and Environmental Science	4	2	1	2018
Trends in Plant Science	3	426	3	2014
Journal of Economic Entomology	3	92	3	1997
Medical and Veterinary Entomology	3	76	3	2008
Phytochemistry Reviews	3	61	3	2011
International Journal of Tropical Insect Science	3	49	2	2006
Ecotoxicology and Environmental Safety	3	31	2	2010
ACS Sustainable Chemistry and Engineering	3	26	2	2016

NP: number of publications; TC: total citations; PY-Start: publication year start.

## Data Availability

Data are available on request to the corresponding author.
